# Influence of Exogenous Melatonin on the Physiological Traits of *Camellia hainanica* Seedlings Under Polyethylene Glycol-Induced Drought Stress

**DOI:** 10.3390/plants14050676

**Published:** 2025-02-22

**Authors:** Liyan Ge, Muhammad Zeeshan Ul Haq, Yanqiang Yao, Dongmei Yang, Ya Liu, Huageng Yang, Yougen Wu

**Affiliations:** School of Breeding and Multiplication (Sanya Institute of Breeding and Multiplication), School of Tropical Agriculture and Forestry, Hainan University, Sanya 572025, China

**Keywords:** *Camellia hainanica*, melatonin (MT), PEG-6000, physiological property

## Abstract

This study investigated the effects of exogenous melatonin (MT) on the physiological responses of *Camellia hainanica* seedlings under drought stress, using the drought-tolerant variety “Hai Da 1” and the drought-sensitive variety “Wan Hai 1” as test materials. Seedlings were treated with MT at concentrations of 0, 50, 100, 150, 200, and 250 μmol/L through irrigation, followed by drought stress induced by polyethylene glycol (PEG-6000). The results revealed that MT alleviated growth damage caused by PEG-simulated drought stress, with leaf relative conductivity and malondialdehyde (MDA) content showing an initial decrease followed by an increase as MT concentration rose. In contrast, relative water content, chlorophyll content, antioxidant enzyme activity, secondary metabolite levels, and carbohydrate content initially increased and then declined with increasing MT concentration. Treatment with 200 μmol/L MT notably reduced MDA content by 40–50%, enhanced antioxidant enzyme activity by 20–30%, and increased secondary metabolite levels by 11–42% in the drought-sensitive variety. These findings identified 200 μmol/L MT as the optimal concentration for mitigating drought stress in *C. hainanica* seedlings, providing a foundation for its practical application in *C. hainanica* production and further research into the drought resistance mechanisms mediated by MT.

## 1. Introduction

In the context of global climate change, drought stress has become a significant environmental challenge that adversely affects plant growth, development, and agricultural productivity [1]. Under drought conditions, plants undergo a series of physiological and biochemical changes, which, in severe cases, can lead to plant mortality [2]. Polyethylene glycol (PEG-6000), a hydrophilic polymer with a high molecular weight, is widely employed to simulate drought stress by manipulating the osmotic pressure of solutions [3]. By adjusting the concentration of PEG-6000, researchers can effectively replicate varying degrees of water deficit conditions [4]. This approach is frequently utilized in studies aimed at improving drought tolerance in plants, with applications in crops such as winter *Triticum aestivum* [5], *Glycine max* [6], *Zea mays* [7], *Juglans regia* [8], and *Malus prunifolia* [9]. The effects of drought stress on plants are often assessed through changes in morphological traits, such as leaf structure [10], and physiological parameters, including photosynthetic pigment levels [11], relative water content [12], secondary metabolite production [13], antioxidant enzyme activity [14], and osmotic regulation [15]. However, *Camellia vietnamensis* Huang has a certain level of drought tolerance. Nonetheless, prolonged dry conditions substantially impede its growth. During extended droughts, this plant’s leaves tend to turn yellow due to reduced chlorophyll levels, and fruit drop increases. These factors ultimately result in diminished yield and quality. Therefore, improving the drought resistance of *C. vietnamensis* from Hainan is essential for sustaining and enhancing its yield and quality [16].

In recent years, there has been an increasing interest in enhancing plant drought resilience by applying exogenous compounds. MT, an emerging plant hormone [17], functions as a signaling molecule and plays a vital role in regulating plant growth and improving stress tolerance [18]. Research has demonstrated that MT, as both a growth regulator and antioxidant, mitigates the detrimental effects of abiotic stress by interacting with other plant hormones [19]. It protects plants from oxidative damage, delays leaf senescence, and enhances antioxidant enzyme activity [20], activating antioxidant defense mechanisms. Under drought stress, MT pretreatment modulates various physiological processes, including osmoregulation [21], photosynthesis [22], senescence, and hormonal crosstalk [23]. For instance, the pretreatment of black *T. aestivum* [24] and *Z. mays* seedlings [25] with different concentrations of MT has been shown to alleviate drought-induced damage, improve reactive oxygen species (ROS) scavenging, enhance energy metabolism, and increase photosynthetic pigment levels, relative water content, and antioxidant enzyme activity. These effects collectively contribute to improved drought tolerance in plants. The current research predominantly centers on crops such as *Z. mays*, *T. Aestivum* [26], and *Gossypium hirsutum* [27]. No studies have reported enhancing drought resistance in *C. hainanica* from Hainan by applying exogenous MT.

*Camellia hainanica*, native to the tropical region of Hainan Island, China [28,29], is a valuable oil tea species renowned for its health benefits, including the prevention of cardiovascular and cerebrovascular diseases [30], cholesterol reduction [31], and anti-inflammatory and detoxifying properties. The long-term consumption of its oil is also associated with promoting longevity [32]. Recognized by the United Nations Food and Agriculture Organization (FAO) as a high-quality edible plant oil [33], *C. hainanica* holds significant economic and nutritional value. However, due to Hainan Island’s tropical monsoon climate and unique geographical location, drought conditions can occur throughout the year, posing a major challenge to the survival and growth of *C. hainanica* [34]. Drought stress is a critical factor limiting its productivity and viability. This study aims to evaluate the impact of MT on the growth of *C. hainanica* and to elucidate the associated physiological responses related to drought resistance under soil drought stress conditions. The results demonstrated that under water-limited soil conditions, optimal MT concentrations significantly improved the drought tolerance of *C. hainanica* seedlings. These findings contribute to the theoretical understanding of MT’s role in enhancing drought resistance and provide a scientific basis for developing and implementing drought-resistant cultivation strategies for *C. hainanica*.

## 2. Results

### 2.1. Effects of Exogenous MT on Growth and Leaf Water Content

**Figure 1:**
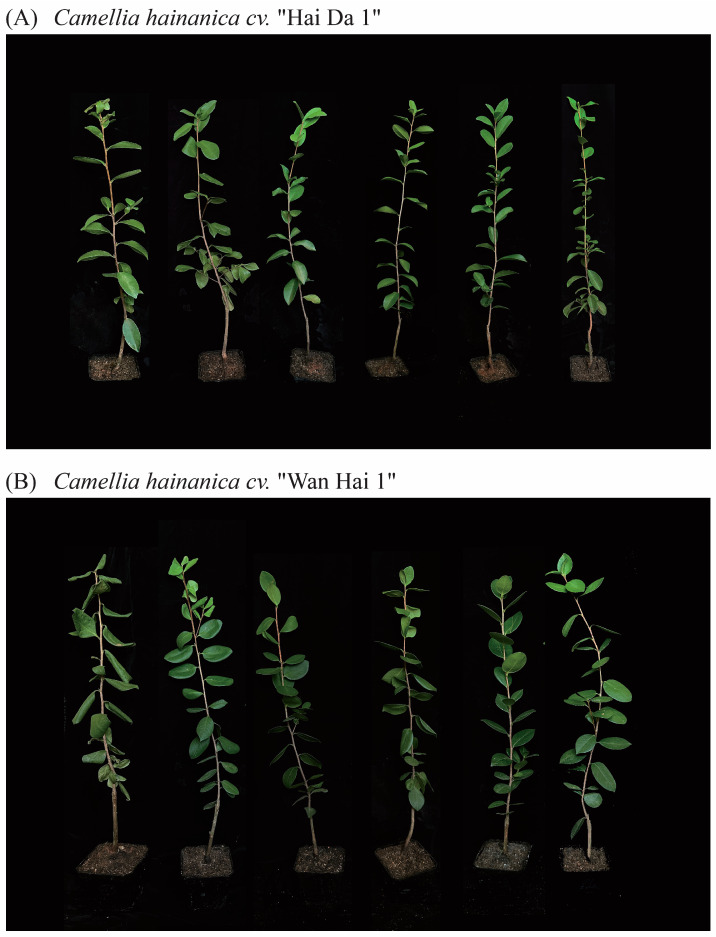
Effect of exogenous MT on *C. hainanica* growth under PEG-induced drought stress. (**A**) The MT pretreatment of drought-stressed “HD 1” on day 12. (**B**) The MT pretreatment of drought-stressed “WH 1” on day 12. Treatments included CK (water + 20% PEG), M1 (50 μmol/L MT + 20% PEG), M2 (100 μmol/L MT + 20% PEG), M3 (150 μmol/L MT + 20% PEG), M4 (200 μmol/L MT + 20% PEG), and M5 (250 μmol/L MT + 20% PEG). Scale bars in the figure are in centimeters.

As the duration of drought stress increased, *C. hainanica* exhibited varying degrees of leaf curling and drooping, which negatively impacted their growth (Figure 1). With higher concentrations of MT, drought-induced damage was alleviated to varying extents in both “HD 1” and “WH 1”. Phenotypically, the alleviation of damage was most pronounced in the M3 (150 μmol/L MT + 20% PEG) and M4 (200 μmol/L MT + 20% PEG) treatments for “HD 1” and the M4 and M5 (250 μmol/L MT + 20% PEG) treatments for “WH 1”. As the MT concentration increased, leaves gradually unfolded, branches exhibited normal growth, and the onset of leaf drooping caused by drought stress was delayed.

**Figure 2:**
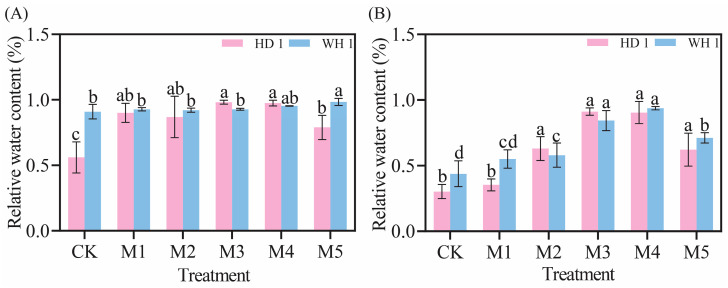
Effect of exogenous MT on leaf relative water content of *C. hainanica* under PEG-induced drought stress. (**A**) The relative water content of leaves on day 6 under MT treatment. (**B**) The relative water content of leaves on day 12 under MT treatment. Different lowercase letters indicate significant differences (*p* < 0.05). Pink bars represent “HD 1”, and blue bars represent “WH 1”.

As illustrated in Figure 2, the relative water content of *C. hainanica* leaves decreased with the prolonged duration of drought stress. However, the response to increasing MT concentrations exhibited an initial increase followed by a subsequent decrease (Figure 2). On day 6 of drought stress under MT treatment (Figure 2A), the relative water content of the leaves of “HD 1” plants decreased. Notably, the relative water content of the leaves in the M3 and M4 treatments increased by 75.36% and 74.10%, respectively, compared to the drought stress group. The relative water content of “WH 1” plants under the M5 treatment increased by 8.25% compared to drought stress. On day 12 of drought stress with MT treatment (Figure 2B), the relative water content of the leaves in both “HD 1” and “WH 1” plants decreased. However, for “HD 1” plants, the relative water content under M3 and M4 treatments increased by 3.01 times and 2.9 times, respectively, while for “WH 1” plants, the relative water content under the M4 treatment increased by 2.14 times compared to the drought stress group.

### 2.2. Effects of Exogenous MT on Leaf Electrical Conductivity

**Figure 3:**
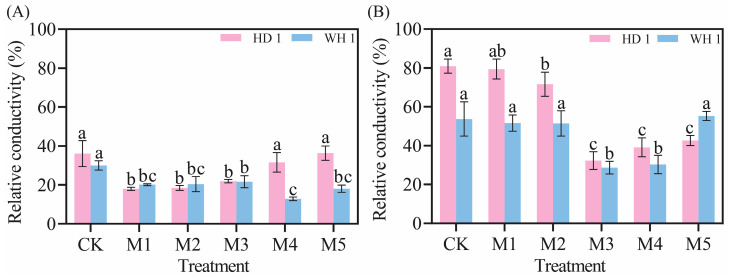
Effect of exogenous MT on relative conductivity of *C. hainanica* under PEG-induced drought stress. (**A**) Relative conductivity of leaves treated with melatonin on day 6 of drought stress. (**B**) Relative conductivity of leaves treated with melatonin on day 12 of drought stress. Different lowercase letters indicate statistically significant differences (*p* < 0.05). Pink bars represent “HD 1”, and blue bars represent “WH 1”.

The relative conductivity of *C. hainanica* leaves increased with prolonged drought stress but exhibited an initial decrease followed by an increase as MT concentration increased (Figure 3). On day 6 of PEG-induced drought stress with MT treatment (Figure 3A), the relative conductivity of “HD 1” leaves in the M1 (50 μmol/L MT + 20% PEG) treatment group decreased by 50.04% compared to the drought stress group, while the relative conductivity of “WH 1” leaves in the M4 treatment group decreased by 57.08%. The relative conductivity showed an upward trend by day 12 of PEG-induced drought stress with MT treatment (Figure 3B). However, the relative conductivity of “HD 1” in the M3 treatment group was 60.14% lower than that of the CK group. In contrast, the relative conductivity of “WH 1” in the M3 and M4 treatment groups decreased by 1.46 times and 1.43 times, respectively, compared to CK.

### 2.3. Effects of Exogenous MT on MDA Levels

**Figure 4:**
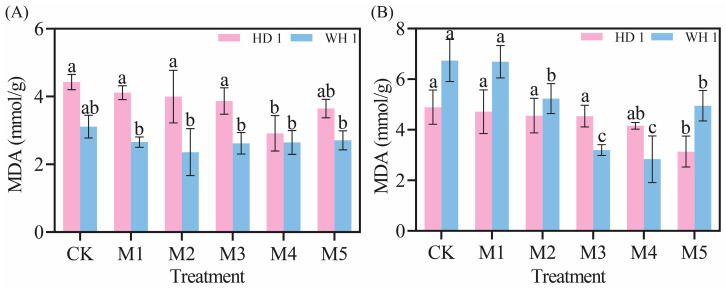
Effects of exogenous MT on MDA content in *C. hainanica* under PEG-induced drought stress. (**A**) MDA content in leaves treated with melatonin on day 6 of drought stress. (**B**) MDA content in leaves treated with melatonin on day 12 of drought stress. Different lowercase letters indicate statistically significant differences (*p* < 0.05). Pink bars represent “HD 1”, and blue bars represent “WH 1”.

As illustrated in Figure 4, the MDA content in the leaves of *C. hainanica* increased progressively with prolonged drought stress. However, as the MT concentration increased, the MDA content initially decreased and then increased again (Figure 4). On day 6 of PEG-induced drought stress with MT treatment (Figure 4A), the M4 treatment group of “HD 1” exhibited a 34.14% reduction in MDA content compared to the CK group, while the M2 (100 μmol/L MT + 20% PEG) treatment group of “WH 1” showed a 24.18% reduction. By day 12 of PEG stress under MT treatment (Figure 4B), the M5 treatment group of “HD 1” demonstrated a 1.35-fold reduction in MDA content compared to the CK group, and the M4 treatment group of “WH 1” exhibited a 1.57-fold reduction, with significant differences observed.

### 2.4. Effects of Exogenous MT on Leaf Chlorophyll Contents

**Figure 5:**
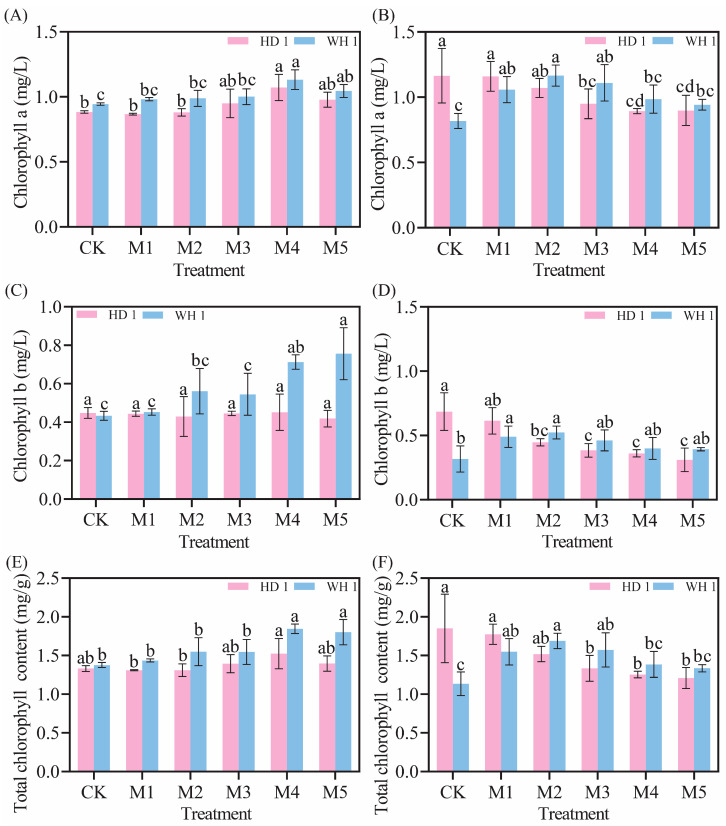
Effects of exogenous MT on chlorophyll a, chlorophyll b, and total chlorophyll content in *C. hainanica* under PEG-induced drought stress. (**A**) Chlorophyll a content treated with MT on day 6 of drought stress. (**B**) Chlorophyll a content on day 12 of MT treatment under drought stress. (**C**) Chlorophyll b content treated with MT on day 6 of drought stress. (**D**) Chlorophyll b content on day 12 of MT treatment under drought stress. (**E**) Total chlorophyll content treated with MT on day 6 of drought stress. (**F**) Total chlorophyll content on day 12 of MT treatment under drought stress. Different lowercase letters indicate statistically significant differences (*p* < 0.05). Pink represents “HD 1”, and blue represents “WH 1”.

The chlorophyll a, chlorophyll b, and total chlorophyll content in the CK group of “HD 1” exhibited an upward trend as drought stress progressed, while in “WH 1”, these parameters showed a declining trend (Figure 5). On day 6 of PEG-induced drought stress (Figure 5A,C,E), compared to the drought stress group, the chlorophyll a, chlorophyll b, and total chlorophyll content in the M4 treatment group of “HD 1” increased by 21.35%, 0.87%, and 14.16%, respectively. In “WH 1”, the M4 treatment group showed increases of 40.36%, 64.71%, and 34.03%, respectively, while chlorophyll b content in the M5 treatment group increased by 74.74%. On day 12 of PEG-induced drought stress (Figure 5B,D,F), the chlorophyll a, chlorophyll b, and total chlorophyll content in the CK group of “WH 1” continued to decrease, whereas these parameters in the M2 treatment group increased by 1.42 times, 1.65 times, and 1.48 times, respectively, with significant differences observed.

### 2.5. Effects of Exogenous MT on Antioxidant Enzymes

**Figure 6:**
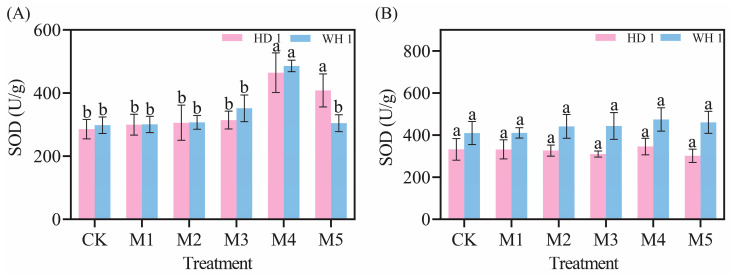
Effects of exogenous MT on SOD activity in leaves of *C. hainanica* under PEG-induced drought stress. (**A**) SOD activity in leaves treated with MT on day 6 of drought stress. (**B**) SOD activity in leaves on day 12 of MT treatment under drought stress. Different lowercase letters indicate statistically significant differences (*p* < 0.05). Pink represents “HD 1”, and blue represents “WH 1”.

As shown in Figure 6, the SOD activity in the leaves of *C. hainanica* gradually increased with the duration of drought stress and also showed a rising trend with increasing MT concentration (Figure 6). On day 6 of PEG-induced drought stress (Figure 6A), SOD activity in the leaves of *C. hainanica* increased significantly. Compared to the CK group, SOD activity in the M5 treatment group increased by 62.60% for “HD 1” and 62.95% for “WH 1”. By day 12 of PEG-induced drought stress (Figure 6B), SOD activity in the M1 treatment group of “HD 1” was 1.04 times higher than that of the CK group. In contrast, SOD activity in the M5 treatment group of “WH 1” was 1.15 times higher than that of the CK group.

**Figure 7:**
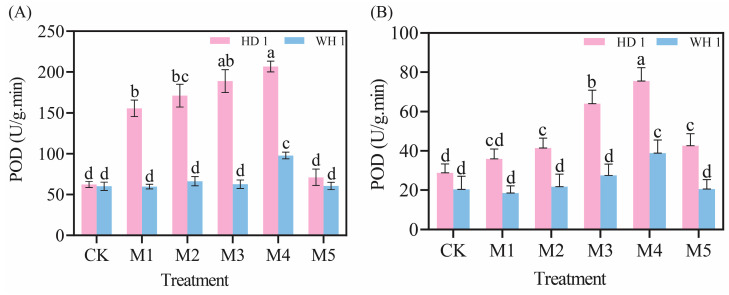
Effects of exogenous MT on POD activity in leaves of *C. hainanica* under PEG-induced drought stress. (**A**) POD activity in leaves treated with MT on day 6 of drought stress. (**B**) POD activity in leaves on day 12 of MT treatment under drought stress. Different lowercase letters indicate statistically significant differences (*p* < 0.05). Pink represents “HD 1”, and blue represents “WH 1”.

The POD activity in the leaves of *C. hainanica* gradually decreased with prolonged drought stress, while it initially increased and then declined with rising MT concentrations (Figure 7). POD activity significantly declined on day 6 of PEG-induced drought stress (Figure 7A). However, compared to the CK group, POD activity in the M1 treatment group of “HD 1” increased by 232.14%, while the M2 treatment group of “WH 1” showed a 61.66% increase. By day 12 of PEG-induced drought stress (Figure 7B), POD activity in the M4 treatment group of “HD 1” increased by 2.61 times, and in the M2 treatment group of “WH 1”, it increased by 1.90 times compared to the CK group.

### 2.6. Effects of Exogenous MT on Secondary Metabolites

**Figure 8:**
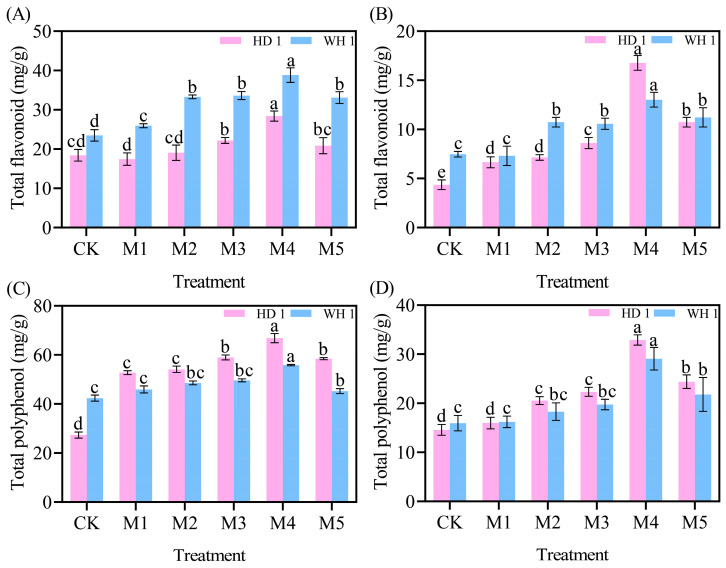
Effects of exogenous MT on secondary metabolites in leaves of *C. hainanica* under PEG-induced drought stress. (**A**) Total flavonoid content in leaves treated with MT on day 6 of drought stress. (**B**) Total flavonoid content in leaves on day 12 of MT treatment under drought stress. (**C**) Total polyphenol content in leaves treated with MT on day 6 of drought stress. (**D**) Total polyphenol content in leaves on day 12 of MT treatment under drought stress. Different lowercase letters indicate statistically significant differences (*p* < 0.05). Pink represents “HD 1”, and blue represents “WH 1”.

The total polyphenol and total flavonoid contents in the leaves of *C. hainanica* progressively decreased with prolonged drought stress while exhibiting a trend of initially increasing and then decreasing with rising MT concentrations (Figure 8). On day 6 of PEG-induced drought stress (Figure 8A,C), the total polyphenol and total flavonoid contents showed a general decline. However, compared to the CK group, the M4 treatment group of “HD 1” showed increases of 54.15% in total polyphenol content and 144.47% in total flavonoid content, whereas the M4 treatment group of “WH 1” demonstrated increases of 65.37% and 31.70%, respectively. By day 12 of PEG-induced drought stress (Figure 8B,D), the levels of secondary metabolites in the CK groups of both “HD 1” and “WH 1” had further decreased. Notably, the total polyphenol and total flavonoid contents in the M4 treatment groups of “HD 1” and “WH 1” increased significantly, by 3.84-fold, 2.25-fold, 1.74-fold, and 1.82-fold, respectively, compared to the CK group.

### 2.7. Effects of Exogenous MT on Carbohydrates

**Figure 9:**
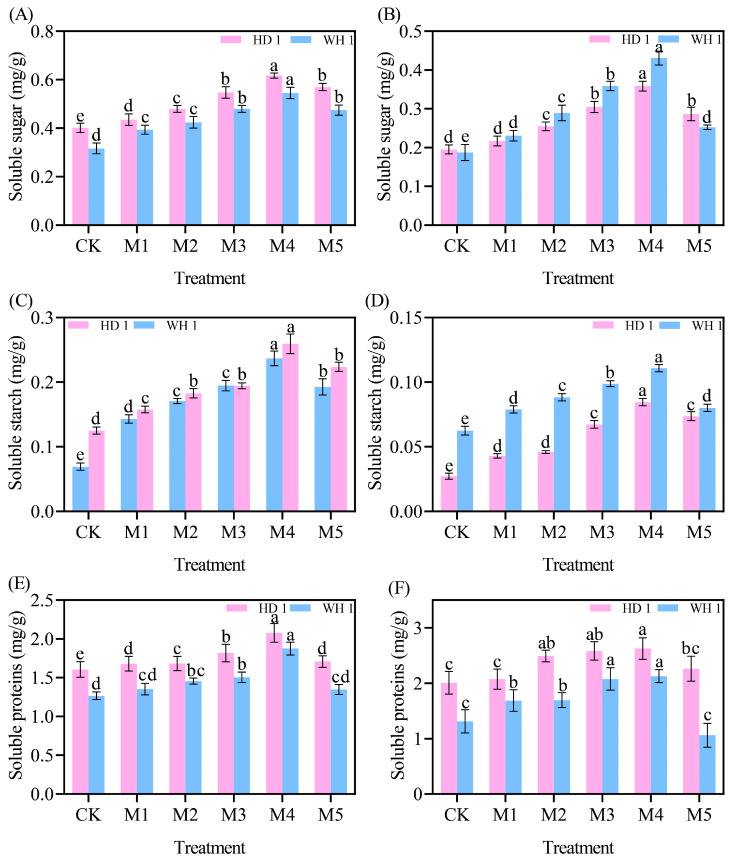
Effects of exogenous MT on leaf carbohydrate content in *C. hainanica* under PEG-induced drought stress. (**A**) Soluble sugar content in leaves treated with MT on day 6 of drought stress. (**B**) Soluble sugar content in leaves on day 12 of MT treatment under drought stress. (**C**) Soluble starch content in leaves treated with MT on day 6 of drought stress. (**D**) Soluble starch content in leaves on day 12 of MT treatment under drought stress. (**E**) Soluble protein content in leaves treated with MT on day 6 of drought stress. (**F**) Soluble protein content in leaves on day 12 of MT treatment under drought stress. Different lowercase letters indicate statistically significant differences (*p* < 0.05). Pink represents “HD 1”, and blue represents “WH 1”.

The contents of soluble sugar and starch in *C. hainanica* leaves exhibited an overall declining trend with the extension of drought stress, whereas the soluble protein content displayed an increasing trend (Figure 9). On day 6 of PEG-induced drought stress (Figure 9A,C,E), the soluble sugar, soluble starch, and soluble protein contents in the M4 treatment group of “HD 1” increased by 52.50%, 108.33%, and 29.37%, respectively, compared to the CK group. Similarly, in the M4 treatment group of “WH 1”, these values increased by 74.19%, 283.33%, and 48.41%, respectively. By day 12 of PEG stress (Figure 9B,D,F), the soluble sugar, soluble starch, and soluble protein contents in the M4 treatment group of “HD 1” increased by 1.84-fold, 3.11-fold, and 1.31-fold, respectively, relative to the CK group. Correspondingly, these values in the M4 treatment group of “WH 1” increased by 2.38-fold, 1.83-fold, and 1.61-fold, respectively.

## 3. Discussion

### 3.1. Effects of MT on the Phenotype of C. hainanica Under Drought Stress

Drought is a major constraint on agricultural productivity, significantly impairing plant growth, development, and yield [35]. The research has demonstrated that melatonin can mitigate drought-induced growth retardation in crops such as cotton, enhancing plant growth and overall health under stress conditions [36]. The findings of this study indicate that drought stress inhibits the normal development of *C. hainanica*, resulting in leaf wilting, curling, and a progressive decline in relative water content over time (Figure 1 and Figure 2). However, irrigation with exogenous MT at varying concentrations effectively increased the relative water content in the leaves of *C. hainanica* by 74–200%, alleviated drought-induced damage, and supported the plants’ ability to regulate water uptake.

### 3.2. Effects of MT on Physiological Indices of C. hainanica Leaves

Under drought stress, plants exhibit an increase in relative conductivity, reflecting an elevated level of cellular damage [37]. Prolonged or intensified drought conditions disrupt the ROS metabolism in plant cells [38], leading to increased membrane lipid peroxidation. MDA, a key end-product of membrane lipid peroxidation, is a critical physiological indicator of stress resistance [39]. Figure 3 and Figure 4 revealed that drought stress significantly increased both 51% relative conductivity and 3.6 mmol/g MDA content in *C. hainanica*, impairing cellular stability and inducing oxidative damage. However, the application of exogenous MT effectively reduced MDA content and relative conductivity, mitigating damage to the membrane system in *C. hainanica* seedlings. This, in turn, decreased the extent of membrane lipid peroxidation, thereby enhancing the drought resistance of *C. hainanica*. Prolonged drought stress inhibits plant growth and disrupts the structural integrity of chloroplasts, particularly the disintegration of thylakoids, which impairs the conversion of light energy into chemical energy [40]. This process leads to a reduction in photosynthetic pigment content in the leaves [41]. The MT has been shown to play a crucial role in regulating plant growth and mitigating the effects of drought stress. Under drought conditions, chlorophyll degradation is a common response. In this study, drought stress significantly reduced chlorophyll content in the leaves of *C. hainanica*, with the extent of reduction closely linked to the duration of drought exposure. These findings align with previous studies on flax (*Linum usitatissimum*) varieties under similar conditions [42]. Under drought conditions, applying MT mitigates the adverse effects of drought stress by enhancing ROS scavenging efficiency, protecting the photosynthetic apparatus, and reducing oxidative damage caused by drought-induced stress [43]. In this study, exogenous MT treatment increased the activities of key antioxidant enzymes by 61–161%, such as SOD and POD, in *C. hainanica* seedlings under drought stress. This enhancement of the antioxidant defense system improved the plant’s ability to cope with drought-induced oxidative damage. However, the protective effect of MT gradually diminished with prolonged exposure to drought stress, a finding consistent with previous studies on *Carya illinoinensis* [44]. Total flavonoids and total polyphenols are vital secondary metabolites with notable antioxidant properties [45]. They play a crucial role in scavenging excessive ROS generated under abiotic stress, thereby protecting plant cells from oxidative damage. Additionally, these metabolites are involved in plant signal transduction pathways and help regulate the plant’s response to drought stress [46]. Total flavonoids, in particular, interact with ROS by donating hydrogen atoms, converting ROS into more stable compounds, which effectively neutralizes free radicals and mitigates oxidative damage to plant cells [47]. In this study, exogenous MT pretreatment significantly increased the total flavonoid content in the leaves of *C. hainanica* under drought stress. By efficiently scavenging free radicals, total flavonoids safeguard the structural integrity of cellular membranes, proteins, and nucleic acids, preserving the normal physiological functions of plant cells. This mechanism contributes to enhanced drought tolerance in *C. hainanica*.

The research has demonstrated that the antioxidant properties of total polyphenols allow them to scavenge ROS directly within cells, thereby minimizing the damaging effects of ROS on cellular membranes [48]. In this study, we observed that MT pretreatment significantly enhanced the total polyphenol content in plant leaves under drought stress. The ROS-scavenging capacity of total polyphenols was positively correlated with the stabilization of cell membranes, by effectively neutralizing ROS, total polyphenols directly protected cell membranes and indirectly safeguarded them by modulating the plant’s antioxidant enzyme system [49]. Total polyphenols can induce the activity of key antioxidant enzymes such as SOD and POD. These enzymes work synergistically to eliminate intracellular ROS, mitigate oxidative stress-induced membrane damage, and ultimately enhance the drought tolerance of *C. hainanica*. Soluble sugars [50], soluble starch [51], and soluble proteins [52] play crucial roles as osmotic adjustment substances, enabling plants to maintain cellular osmotic balance. Under drought conditions, water scarcity reduces cellular water potential and increases osmotic stress [53]. Drought stress disrupts starch metabolism and protein structure, impairing their functionality. The MT mitigates these effects by increasing the content of soluble sugars, stabilizing soluble starch levels, and promoting the synthesis of soluble proteins. This enhances cellular osmotic adjustment, reduces intracellular water potential, and facilitates water uptake from the surrounding environment. MT strengthens plant stress resistance by maintaining cell turgor and supporting normal physiological functions, providing a foundation for recovery and growth following drought conditions.

## 4. Materials and Methods

### 4.1. Plant Materials and Growth Conditions

In this study, “Hai Da 1” (a drought-tolerant variety, “HD 1”) and “Wan Hai 1” (a drought-sensitive variety, “WD 1”) from Vietnam were used. The plants were grown in plastic pots with a diameter of 10 cm and a depth of 8.6 cm, each containing approximately 0.2 L of nutrient soil. The nutrient soil consisted of a 3:1 mixture of peat and vermiculite. All seedlings were grown in a controlled greenhouse at the Hainan University Research Institute of Breeding and Multiplication (N 18°09′34″, E 108°56′30″) in Yazhou, Hainan, China. The greenhouse conditions were maintained at 25 ± 2 °C with 60% relative humidity, a 16 h light/8 h dark photoperiod, and a light intensity of 2000 lux.

### 4.2. Experimental Design and Treatment Management

After approximately three months of standard cultivation, uniform seedlings were selected for experimental treatments. Starting at 18:00 on alternate days, 80 mL of MT solution at various concentrations (0, 50, 100, 150, 200, and 250 μmol/L) was applied to seedlings of both *C. hainanica* varieties, totaling five applications. Subsequently, 100 mL of a 20% PEG solution was administered to each pot every other day to simulate drought stress conditions, with no additional liquid applied during the treatment period. Samples were collected at 10:00 AM on days 6 and 12 after the onset of drought stress. For each treatment group at each sampling time point, three biological replicates were randomly selected, rapidly frozen in liquid nitrogen, and stored at −80 °C for further analysis. The experimental groups were as follows: CK (control group, irrigated with clear water + 20% PEG), M1 (irrigated with 50 μmol/L MT + 20% PEG), M2 (irrigated with 100 μmol/L MT + 20% PEG), M3 (irrigated with 150 μmol/L MT + 20% PEG), M4 (irrigated with 200 μmol/L MT + 20% PEG), and M5 (irrigated with 250 μmol/L MT + 20% PEG).

### 4.3. Physiological Indexes Measurement

The relative water content of leaves was determined using the saturated water content method, while chlorophyll content was measured using the acetone extraction method [54]. Relative electrical conductivity was assessed with a conductivity meter (DDSJ-308A-Intelligent conductivity instrument), and malondialdehyde (MDA) content was quantified using the thiobarbituric acid (TBA) method [55].

A total of 0.10 g of freshly harvested seedlings was homogenized in 1 mL of phosphate buffer (50 mM, pH of 7.8). The homogenate was centrifuged at 12,000× *g* for 20 min at 4 °C, and the supernatant was collected for enzyme activity assays. Peroxidase (POD) activity was evaluated using the guaiacol chromogenic method, and superoxide dismutase (SOD) activity was measured based on the nitroblue tetrazolium (NBT) photoreduction method [56].

For test samples of *C. hainanica*, 1 mL of each sample solution was placed in a 10 mL brown volumetric flask, diluted 10 times with distilled water, and brought to volume. After thorough mixing, 1 mL of this solution was transferred for analysis, and total flavonoid and polyphenol contents were determined using UV–visible spectrophotometry [57].

A 0.1 g sample was put into 5 mL pure water and extracted with boiling water for 30 min (repeated twice), and the volume was fixed to 25 mL to wait for the determination of soluble sugar. The remaining residue was used to determine soluble starch. Soluble protein content was quantified using the Coomassie Brilliant Blue G-250 staining method [58].

### 4.4. Statistical Analysis

Data analysis was conducted using SPSS 25.0 (IBM, Armonk, NY, USA), with Duncan’s multiple range (DMR) test applied for significance analysis (*p* < 0.05). Graphical representation of the results was performed using GraphPad Prism 8.0, with values presented as the mean ± standard deviation of three biological replicates.

## 5. Conclusions

In conclusion, applying exogenous MT mitigated the adverse effects of drought stress on *C. hainanica* seedlings. MT increased the chlorophyll and relative water content, reducing water loss and delaying wilting under drought conditions. The MT enhanced the activities of antioxidant enzymes, facilitated the scavenging of ROS, maintained the redox balance within leaf cells, reduced relative conductivity and MDA content, and alleviated the degradation of secondary metabolites such as total flavonoids and total polyphenols caused by drought stress. Furthermore, it elevated the soluble sugars, starch, and protein levels in *C. hainanica* leaves, providing essential energy and substrates for post-drought recovery and growth. Overall, MT pretreatment significantly improved the drought tolerance of *C. hainanica* seedlings. Among the tested concentrations, 200 μmol/L MT exhibited the most effective alleviation of drought-induced stress. This study provides a theoretical foundation for the development and utilization of MT and contributes to the genetic improvement of drought tolerance in plants under arid environmental conditions by analyzing the physiological mechanisms through which melatonin alleviates drought stress in *C. hainanica*.

## Data Availability

Data will be made available on request.
